# Supplementary material for: Periodically correlated time series and the Variable Bandpass Periodic Block Bootstrap
Source: PLoS One. 2024 Sep 17;19(9):e0310563. doi: 10.1371/journal.pone.0310563 (PMC11407666; doi:10.1371/journal.pone.0310563)
Supplement: S1 Appendix — (PDF) [file pone.0310563.s001.pdf]

```
## R code:
# Generates a PC component with period pcperiod=100 of length xtot=1000.
pcperiod=100
xtot=1000
pc.v <- array(NA, dim = xtot)
for (i in 1:xtot)
{pc.v[i] <- sin(2*pi*((i-1)/pcperiod))}
# Generates an error component from random draws of a N(mean=0,var=2).
et.v<- rnorm(xtot,0,sqrt(2))
# Creates the simulated PC time series composed of the PC and noise components.
data.v <- pc.v + et.v

## PBB bootstraps the PC time series with blocks of length p.
## Generates a matrix pbb.m of B bootstraps where each row is one PBB resample.
temp.v<-data.v
B=1000
p=100
pbb.m<-matrix(NA, B, xtot)
for (k in 1:p)
{extract.temp.v<-temp.v[seq(k,length(temp.v),p)]
for (i in 1:B)
{pbb.m[i,seq(k,length(temp.v),p)]<-
sample(extract.temp.v,size=length(extract.temp.v), replace=TRUE)}}

## VBPBB bootstraps the KZFT (with arguments f=1/p, m=11, k=1) bandpass
## filtered PC component with blocks of length p.
## Generates a matrix vbpbb.m of B bootstraps where each row is one VBPBB
## resample.
library(kza)
kzftpc<-kzft(data.v,m=11,k=1,f=1/p)
kzftpc.v<-2*Re(kzftpc)
B=1000
p=100
temp.v <- kzftpc.v
vbpbb.m<-matrix(NA, B , xtot )
for (k in 1:p)
{extract.temp.v<-temp.v[seq(k,length(temp.v),p)]
for (i in 1:B)
{vbpbb.m[i,seq(k,length(temp.v),p)]<-
sample(extract.temp.v,size=length(extract.temp.v), replace=TRUE)}}
```
